# Astrocyte responses to experimental glaucoma in mouse optic nerve head

**DOI:** 10.1371/journal.pone.0238104

**Published:** 2020-08-21

**Authors:** Sarah Quillen, Julie Schaub, Harry Quigley, Mary Pease, Arina Korneva, Elizabeth Kimball

**Affiliations:** Glaucoma Center of Excellence, Wilmer Ophthalmological Institute, Johns Hopkins University, Baltimore, Maryland, United States of America; Instituto Murciano de Investigacion y Desarrollo Agrario y Alimentario, SPAIN

## Abstract

**Purpose:**

To delineate responses of optic nerve head astrocytes to sustained intraocular pressure (IOP) elevation in mice.

**Methods:**

We elevated IOP for 1 day to 6 weeks by intracameral microbead injection in 4 strains of mice. Astrocyte alterations were studied by transmission electron microscopy (TEM) including immunogold molecular localization, and by laser scanning microscopy (LSM) with immunofluorescence for integrin β1, α-dystroglycan, and glial fibrillary acidic protein (GFAP). Astrocyte proliferation and apoptosis were quantified by Ki67 and TUNEL labeling, respectively.

**Results:**

Astrocytes in normal optic nerve head expressed integrin β1 and α-dystroglycan by LSM and TEM immunogold labeling at electron dense junctional complexes that were found only on cell membrane zones bordering their basement membranes (BM) at the peripapillary sclera (PPS) and optic nerve head capillaries. At 1–3 days after IOP elevation, abnormal extracellular spaces appeared between astrocytes near PPS, and axonal vesical and mitochondrial accumulation indicated axonal transport blockade. By 1 week, abnormal spaces increased, new collagen formation occurred, and astrocytes separated from their BM, leaving cell membrane fragments. Electron dense junctional complexes separated or were absent at the BM. Astrocyte proliferation was modest during the first week, while only occasional apoptotic astrocytes were observed by TEM and TUNEL.

**Conclusions:**

Astrocytes normally exhibit junctions with their BM which are disrupted by extended IOP elevation. Responses include reorientation of cell processes, new collagen formation, and cell proliferation.

## Introduction

Glaucoma injury to retinal ganglion cells (RGC) is initiated at the optic nerve head (ONH) in experimental animal models of acute [1] or chronic intraocular pressure (IOP) elevation [2–4], as it is in human glaucoma eyes [5]. One major cause of RGC injury and death is IOP-generated mechanical stress, manifesting both as hoop stress on the ONH at the peripapillary sclera (PPS) and as the translaminar pressure difference between IOP and optic nerve (ON) tissue pressure. The extracellular fiber structure and orientation, as well as the cellular orientation of the PPS and ONH are designed to withstand these stresses. Collagen and elastic fibers in the PPS are arranged circumferentially around the ONH in mammalian eyes [6–8]. Astrocytes make up the rodent lamina cribrosa (LC) [9] which is largely cellular, while the eyes of larger mammals have astrocyte-covered connective tissue beams comprising the LC. Both the astrocytic LC of mice and rats, and the beams of larger animal LC, are arrayed directly across the ONH from one side to the other [10, 11]. Collagen fibrils within the human LC are smaller and more uniform in diameter than those of the sclera, probably to maximize resistance to mechanical stress [12].

Regional differences within both rodent astrocytic LC and large mammal, beam-containing LC are associated with selectively greater injury and RGC death for axons passing through particular ONH zones in glaucoma [13–15], with more susceptible areas showing greater strain with IOP increase [16]. These early mechanical events are followed by remodeling of ONH and PPS structure, directed by PPS fibroblasts and ONH astrocytes (and LC fibroblasts in animals with ONH connective tissue beams). The pathways operative in the early remodeling process are not fully understood and are the subject of the present report. In terms of overall structural changes, human glaucoma leads to widening of the ONH behind Bruch’s membrane [17, 18] along with LC beam compression [19, 20]. Early reorganization that can be studied in monkey glaucoma models has identified an immediate, hypercompliant mechanical response to IOP elevation [21–23]. This is followed later by an increase in overall and regional stiffness in monkey [24], human [25, 26], and mouse [27] eyes. It has not been established what cellular or extracellular events underlie these two phases of ONH response.

The mechanosensing mechanisms of astrocytes [28] are likely vital elements in both protective and detrimental effects of IOP-generated stress. Astrocytes maintain homeostasis in the zone surrounding axons and are important in transmitting and mitigating mechanical and biochemical stress for RGC axons. Our proteomic assay of mouse posterior sclera and ONH tissues found that molecules specifically upregulated by chronic experimental IOP increase are characteristic of actin—cytoskeleton signaling, integrin-linked signaling, and Rho-kinase signaling [29]. Further clarification is needed for the mechanisms underlying transduction of astrocyte mechanosensation of IOP-generated stress into cellular responses. While this area is critically important, even the specific membrane-bound molecular linkages between normal ONH astrocytes and their basement membrane (BM) were not known until recently. In the present investigations, the junctional complexes joining astrocytes to their BM at the scleral canal are delineated and studied after extended IOP elevation. We detail the regional phenotypic characteristics of astrocytes in the unmyelinated ONH where glaucoma injury occurs, especially at the PPS, a primary zone where mouse ocular astrocytes contact the connective tissue matrix and may be affected by changes in scleral stress.

## Methods

### Animals

All animals were treated in accordance with the guidelines of the ARVO Statement for the Use of Animals in Ophthalmic and Vision Research, using protocols approved and monitored by the Johns Hopkins University School of Medicine Animal Care and Use Committee. Different strains of mice, 2–6 months of age at the start of experiments were studied: CD1 (Charles River Laboratories, Wilmington, MA, USA), C57BL/6 (B6, Jackson Laboratories, Bar Harbor, ME, USA), GLT1-GFP [30] (GLT1/eGFP/Bac are a transgenic strain of mice expressing green fluorescent protein, GFP, under control of the glutamate transporter 1 (GLT1) promoter, B6 background, courtesy of Jeffrey Rothstein, Johns Hopkins School of Medicine, Baltimore, MD, USA) and GFP-GFAP [31] (FVB/N-Tg(GFAPGFP)14Mes/J are a transgenic strain of mice expressing green fluorescent protein (GFP), under the glial fibrillary acidic protein (GFAP) promoter, FvB background, commercially available through Jackson Laboratories, Bar Harbor, ME, USA). The 4 types of mice were included to determine if the findings were essentially similar in albino, agouti and pigmented mice. We have published data on each of these strains and each has important strengths for particular experimental protocols. For example, the mice with fluorescent (GFP) astrocytes were previously used in biomechanical studies and CD1 albino and B6 pigmented mice were compared in neuroprotection studies. We used the 4 strains to document that the typical findings in astrocyte structure were similar across strains.

In this study, 168 mice were used to study structure either by transmission electron microscopy (TEM) or by immunolabeling after cryopreservation. For TEM, we studied the following number of control, and fellow bead injected eyes: 32 CD1, 44 GLT1-GFP, and 32 GFP-GFAP and bilaterally naïve eyes: 4 GLT1-GFP, and 4 GFP-GFAP. At each of the following time points, we studied 8 eyes from each of the 3 strains of mice after IOP elevation: 1 day, 3 days, 1 week and 6 weeks. Immunogold labeling of specific molecules for TEM was performed in 24 eyes at 1 week after glaucoma induction and their fellow eyes in GLT1-GFP mice. Immunolabeling for TUNEL, ki67, GFAP, α-dystroglycan, integrin β1, as well as phalloidin staining of actin, were carried out in 31 CD1 mice, 22 B6 mice, and 3 GLT1-GFP mice in control and IOP elevated tissue: 1 day, 3 days, 1 week and 6 weeks. Sections of the eyes were imaged by laser scanning microscopy (LSM) (See Table 1).

**Table 1:** Number of mice used by experiment and strain.

Strain	TEM	Immunogold TEM	Immunofluorescence
CD1	Naïve- 0		3 Day Glaucoma-23
	1 Day Glaucoma- 8		1 Week Glaucoma- 4
	3 Day Glaucoma-8	N/A	6 Week Glaucoma- 4
	1 Week Glaucoma-8		
	6 Week Glaucoma-8		
GLT1-GFP	Naïve- 2	1 Week Glaucoma- 12	3 Day Glaucoma-3
	1 Day Glaucoma- 8		
	3 Day Glaucoma-8		
	1 Week Glaucoma-8		
	6 Week Glaucoma-8		
GFP-GFAP	Naïve- 2	N/A	N/A
	1 Day Glaucoma- 8		
	3 Day Glaucoma-8		
	1 Week Glaucoma-8		
	6 Week Glaucoma-8		
B6	N/A	N/A	3 Day Glaucoma-17
			1 Week Glaucoma- 5

* Contralateral eyes were controls.

### Anesthesia

For anterior chamber microbead injections and euthanasia, mice were anesthetized by intraperitoneal injection of ketamine (50 mg/kg, Fort Dodge Animal Health, Fort Dodge, IA), xylazine (10 mg/kg, VedCo Inc., Saint Joseph, MO) and acepromazine (2 mg/kg, Phoenix Pharmaceuticals, Burlingame, CA), along with topical anesthesia eye drops (0.5% proparacaine hydrochloride, Akorn Inc. Buffalo Grove, IL, USA). For IOP measurements only, animals were anesthetized using a Rodent Circuit Controller (VetEquip, Inc., Pleasanton, CA, USA) delivering 2.5% of isoflurane in oxygen, 500cc/minute.

### Intraocular pressure measurement

IOP was measured using the TonoLab rebound tonometer (TioLat, Inc., Helsinki, Finland), recording the mean of 6 readings with optimal quality grading. For bead injected animals, IOP was measured prior to injection, at 1 day, 3 days, 1 week, and 6 weeks after injection, depending upon length of survival.

### Bead injection protocol

Ninety-six mice were anesthetized as described and as previously published [32], the left anterior chamber was injected with Polybead Microspheres (Polysciences, Inc., Warrington, PA, USA), consisting of 2 μL of 6 μm diameter beads, then 2 μL of 1 μm diameter beads, followed by 1 μL of viscoelastic compound (10 mg/ml sodium hyaluronate, Healon; Advanced Medical Optics Inc., Santa Ana, CA). The contralateral eye was used as control.

### Tissue preservation for transmission electron microscopy

Animals for TEM studies were euthanized by exsanguination under general intraperitoneal anesthesia, followed by intracardiac perfusion for 3 minutes with 4% paraformaldehyde in 0.1 M sodium phosphate buffer (Na_3_PO_4_, pH = 7.2), 1 minute of 0.1M cacodylate buffer, and 7 minutes of 2% paraformaldehyde/2.5% glutaraldehyde in cacodylate buffer. A cautery mark on the superior cornea provided subsequent orientation. Eyes were enucleated and kept in fixative overnight. In some eyes, the ON was removed 1.5mm behind the globe and separately processed, while in other eyes globes were processed with their proximal ON attached for epoxy embedding. Tissue was post-fixed in 1% osmium tetroxide, dehydrated in ascending alcohol concentrations, and stained in 1% uranyl acetate in 100% ethanol for 1 hour. Tissues were embedded in epoxy resin mixture at 60°C for 48 hours. One micron thick sections were cut and stained with 1% toluidine blue. Ultra-thin sections (~68nm thick) were collected on copper grids.

### Tissue preservation for laser scanning and light microscopy.

Animals were euthanized as above, with cardiac perfusion for 10 minutes using 4% paraformaldehyde in 0.1 M sodium phosphate buffer (Na_3_PO_4_, pH = 7.2). Eyes were enucleated and post-fixed in 4% paraformaldehyde for 1 hour, then placed into phosphate buffer. The anterior globe and lens were removed. ONs were cut 1.5mm behind the globe and were embedded in epoxy for nerve axon counting in 1 μm sections

For LSM, posterior poles were cryopreserved in sucrose and optimal cutting temperature compound (OCT; Sakura Finetek USA. Inc., Torrance, CA), cryosectioned (in either cross or longitudinal orientation) between 8 to 16 μm thick. Primary antibodies were used at a 1:200 dilution, while secondary antibodies were completed at a 1:500 dilution (Tables 2 and S1). Images were collected on the Zeiss LSM 710 or the Zeiss LSM 510 (Carl Zeiss Microscopy, LLC, Thornwood, NY) using the 63x 1.40 NA Plan Apochromat.

**Table 2:** Table of primary and secondary antibodies.

**Stain**	**Dye**	**Dilution**	**Company (Catalog #)**
DAPI (4',6-Diamidino-2-Phenylindole, Dihydrochloride)	Fluor Dye 405	1:1000	Invitrogen (D1306)
Actin (Phalloidin)	Fluor Dye 568	1:80	Invitrogen (A12380)
TUNEL (In Situ Cell Death Detection Kit)	Fluorescein	Kit	Sigma (11684795910)
**Primary Antibody**	**Species**	**Dilution**	**Company (Catalog #)**
Anti-Integrin β1	Rabbit	1:200	Abcam (AB183666)
Anti-α-dystroglycan	Mouse (IgM)	1:200	EMD Millipore (05–593)
Anti-glial fibrillary acidic protein (GFAP)	Chicken	1:200	Abcam (AB4674)
Anti-myelin basic protein (MBP)	Rabbit	1:200	Abcam (AB218011)
Anti-Ki67	Rabbit	1:200	Abcam (AB15580)
**Secondary Antibody**	**Species, anti-species**	**Dilution**	**Company (Catalog #)**
Laser 488	Goat anti-Rabbit	1:500	Invitrogen (A11008)
Laser 488	Goat anti-mouse (IgM)	1:500	Invitrogen (A21042)
Laser 647	Goat anti- Chicken	1:500	Invitrogen (A32933)

**Fig 1:**
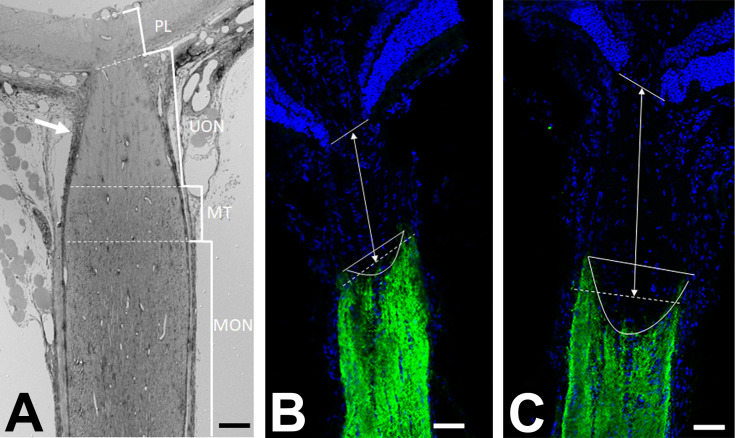
Zones of the mouse optic nerve head region. A: Epoxy embedded section of the optic nerve head, in longitudinal orientation, from a GLT1-GFP mouse, was stained with toluidine blue and imaged in light microscopy. Marked brackets indicate the regions of interest; prelamina (PL), unmyelinated nerve (UON), myelin transition zone (MT), myelinated nerve (MON). Note that the sclera in mouse has a portion that directly joins the UON and a posterior portion that joins with the pia mater (arrow). B,C: Longitudinal cryopreserved sections of the optic nerve in CD1 mice, immunolabeled for DAPI (blue) and antibody to myelin basic protein (MBP, green), shows the delineation between the unmyelinated and myelinated regions of the optic nerve. Compared to position of MBP line in normal nerve (B), it was shifted significantly distal to the eye after 3 day IOP elevation (C). Scale bar = 50 μm (A) 100 μm (B, C).

We used LSM observations to specify the exact location of ONH regions, measuring the distance from Bruch’s membrane opening to the zone at which myelin begins in the optic nerve before and after chronic IOP elevation (Fig 1B and 1C). For this, 18 sections were labeled from 18 mice with anti-myelin basic protein (MBP). Using a Zeiss LSM 710 confocal microscope, a masked investigator measured the distance from Bruch’s membrane opening to the myelin line, taking the average of the minimum and maximum distances.

LSM imaging was used to study astrocyte proliferation by Ki67 labeling and astrocyte death by apoptosis estimated by TUNEL assay. Following cryopreservation as above, sections from CD1, C57BL/6 and GLT1-GFP animals (in the following groups: control, 1 day, 3 day, 1 week and 6 weeks) were incubated overnight with anti-Ki67. The control and glaucoma optic nerve sections were imaged with a LSM 710 using a 20x objective lens. The longitudinal optic nerve was divided into 4 regions; pre-lamina (PL), unmyelinated optic nerve (UON), myelin transition zone (MT), and the myelinated optic nerve (MON). The area of each region was traced using Metamorph Imaging Software (Molecular Devices, Sunnyvale, CA, USA). All Ki67 positive cells within each of the regions were counted to calculate number of positive cells/mm^2^. Terminal deoxynucleotidyl transferase catalyzes the template-independent polymerization of deoxyribonucleotides to the 3′-end of single- and double-stranded DNA. An assay for this event was performed using the In Situ Cell Death Detection Kit (Roche, Basel, Switzerland). Sections from control and glaucoma tissue were sampled for TUNEL positive cells, and nuclei were also stained with DAPI.

Cryopreserved tissue was also sectioned in cross orientation to visualize the astrocytic lamina process network. Slides from GFP-GLT1 mice were imaged using the fluorescent astrocytes, while some samples were stained with phalloidin to delineate actin and with DAPI (Table 2).

#### Optic nerve counts

One μm thick cross-sections of the ON were cut and stained with toluidine blue. Using a Cool Snap camera and Metamorph Image Analysis software low power (10x objective), digital images of the nerves were used to measure each ON area, then high power (100x objective) images were taken to measure and count the axonal fibers. For each ON, five 40 x 40 μm fields were acquired at 100x, corresponding to a 9% sample of total ON area. Masked observers edited non-axonal elements from each image, generating an axon density from the software. The average axon density/mm^2^ was multiplied by the individual nerve area to estimate overall axon number. Experimental eyes were compared to the mean axon number in pooled, fellow eye nerves of the appropriate mouse strain, age, and tissue fixation to yield percent axon loss.

#### Tissue preparation for transmission electron microscopy (TEM)

One μm thick epoxy sections were cut in retina and optic nerve tissues either perpendicular to the optic axis (cross-sections) or parallel to it (longitudinal sections) and stained with 1% toluidine blue. Sections were imaged using Axioscope and Imaging Software (Carl Zeiss Microscopy, LLC White Plains, NY) at 10-63x. Ultrathin sections (~68 nm) were placed on copper grids and stained with uranyl acetate and lead citrate before being examined with a Hitachi H7600 transmission electron microscope (Hitachi High Technologies, Clarksburg, MD). For identification of specific molecules, antibodies fused to gold particles were used after the following protocol. Prior to epoxy embedding, eyes were fixed in 0.2% glutaraldehyde, 4% paraformaldeyde in 0.1M phosphate buffer, pH 7.4 for 3 hours after enucleation, washed in 0.15 mM Sorensen’s phosphate buffer, then stored overnight. The eyes were treated with 0.1% sodium borohydride to deactivate the aldehydes, then placed in Aurion goat blocking solution (Wageningen, The Netherlands), washed and incubated overnight in primary antibody: anti-integrin β1 and anti-α-dystroglycan at 4°C. After repeated washes in PBS, they were incubated with 10 nm gold-conjugated secondary antibody (Wageningen, The Netherlands). Eyes were then post-fixed in 2.5% glutaraldehyde followed by 0.5% OsO4. After dehydration in ascending alcohol concentrations, they were placed in 1% uranyl acetate in 100% ethanol for 1 hour. Tissues were embedded in epoxy resin mixture at 60°C for 48 hours and ultrathin sections cut.

#### Statistical methods

Statistical comparisons between data on IOP elevation, Ki67 labeling, and optic nerve counts used t-tests, with statistical significance indicated at p<0.05, using the commercial software in Microsoft Excel (Microsoft, Redmond, WA).

## Results

### Intraocular pressure

Mean IOP levels were significantly higher after bead injection for each strain of mouse. Compared to baseline control mean IOP = 12.1 ± 3.1 mm Hg and 13.1 ± 3.3 mm Hg (right and left eyes, respectively), the mean IOP at 1 day in bead-injected eyes was 24.0 ± 5.6 mm Hg; 17.9 ± 5.6 mm Hg at 3 days, declining to 14.7 ± 5.2 at 1 week and 14.7 ± 4.6 at 6 weeks (Table 3).

**Table 3:** Mean IOP difference of glaucoma minus control, fellow eyes.

			Time after IOP elevation			
	Mean Baseline IOP	Baseline RE VS LE	1 Day	3Day	1 Week	6 Week
	Mean IOP	Mean diff + STDV	Mean diff + STDV	Mean diff + STDV	Mean diff + STDV	Mean diff + STDV
GFP-GFAP	12.00±3.37	0.88±1.70	10.56±7.54***	6.04±6.32**	1.65±4.72	1.38±4.63
GLT1-GFP	12.60±3.11	1.10±3.60	10.94±6.84***	4.91±7.86*	0.76±6.15	0.67±6.61
CD1	11.75±2.94	1.00±3.07	11.59±5.00***	5.50±6.00*	5.50±7.78*	2.67±7.01
All Strains	12.11±3.13	0.98±2.85	11.03±6.49***	5.27±6.68***	2.69±6.68	1.71±6.05
Total N	95	95	24	23	24	24

Data are mean ± standard deviation (STDV) or mean and STDV of mean difference (diff) in intraocular pressure (IOP) between glaucoma eye and fellow, control eye. Mean baseline IOP is average of two eyes of each mouse.

* p<0.05,

** p<0.01,

***p<0.001, t tests.

### Zones of ONH region

The terminology for zones of the mouse ONH region used in epoxy embedded tissue are: prelaminar (PL: nerve head overlying Bruch’s membrane opening and anterior to Bruch’s membrane), unmyelinated optic nerve (UON: from Bruch’s membrane to the start of myelin line, including zone contacting the choroid and PPS), the myelin transition zone (MT), and myelinated optic nerve (MON, Fig 1A). Note that the mouse sclera both contacts the unmyelinated ONH and divides into an outer portion that is continuous with the pia mater, thus transmitting stress to much of the unmyelinated zone and producing axonal transport blockade with IOP elevation throughout this area.

### Normal ONH structure

**Fig 2:**
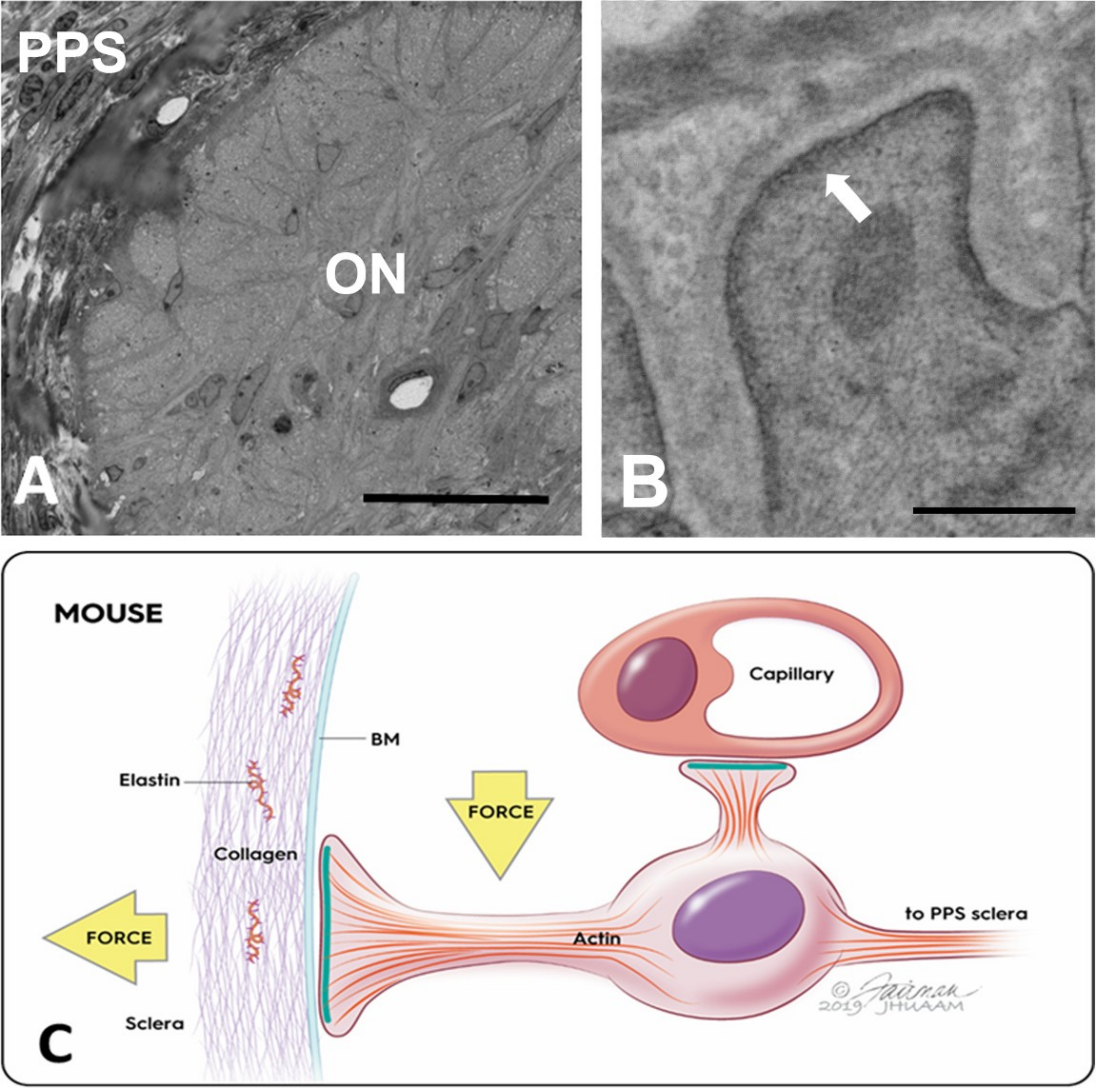
Ultrastructure of astrocyte junctional complexes. A: Epoxy stained cross section in the unmyelinated, normal GFP-GFAP mouse optic nerve (ON), where astrocytes are seen reaching the peripapillary sclera (PPS); B: Transmission electron microscopic image of bilaterally naïve GFP-GFAP mouse ON region with electron dense complexes facing the astrocytic basement membrane (BM) (arrow); C: Schematic showing junctional complex densities (green bands) along inner cell membrane of astrocyte (purple nucleus) only facing BM at PPS and near an optic nerve capillary, but not in the remainder of the astrocyte. Forces acting on astrocyte are both hoop stress from PPS (horizontal yellow arrow) and translaminar pressure gradient (vertical yellow arrow). Scale bars: A = 20 μm, B = 500 nm.

The normal ONH is composed of RGC axons, astrocytes, capillaries with minimal surrounding connective tissue, and microglia. The UON is bounded by astrocytes that secrete a BM separating them and the other cellular components from the choroid, sclera, and pia mater. Wherever astrocytes contact connective tissues, there is continuous BM and on the internal astrocyte cytoplasm facing the BM is an electron dense, junctional complex (Fig 2).

**Fig 3:**
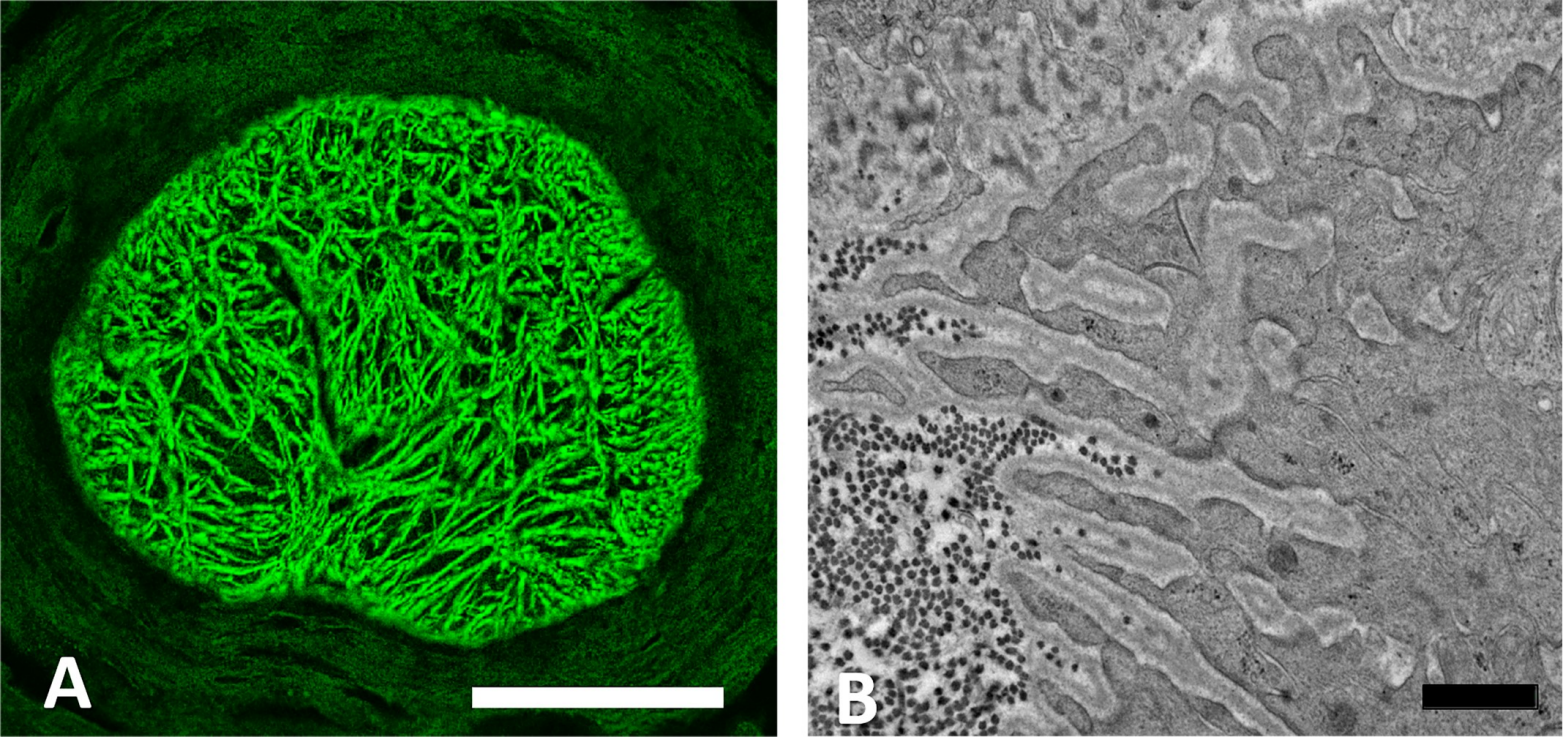
Astrocyte processes in unmyelinated nerve head. A: Laser scanning micrograph of astrocyte processes in the unmyelinated optic nerve (UON) of a normal GLT1-GFP mouse labelled by anti-GFAP antibody (green), showing general pattern of side-to-side processes. B: TEM section at the choroidal—scleral interface in the UON of a normal CD1 mouse showing heavily interdigitated astrocyte processes typical for this location. Basement membrane (BM) is seen within the interdigitations and cross-sectioned dark collagen fibrils are seen in the sclera. Scale bar A = 100 μm; Scale bar B = 1 μm.

Furthermore, axons are segregated from all other nerve head components by fine astrocyte processes. The cell soma and nucleus of astrocytes are arranged in columns in the PL, but from Bruch’s membrane through the UON, each astrocyte is adherent to the PPS on either side, with nuclei at intervals in between axonal bundles, as reported in the human nerve head [11]. The larger astrocyte processes all course laterally from one side of the PPS to the other [9], perpendicular to the course of axons (Fig 3). The PPS contains collagen, elastin, and matrix, as well as fibroblasts, with fibers and cells being circumferentially arranged around the nerve head (Fig 2A). In the mouse, there is an inferior PPS zone through which major retinal blood vessels pass into the ONH and through it to become retinal arteries and veins. The PPS divides as it approaches the nerve head into an inner segment that directly adjoins the astrocytes lining the UON and an outer segment that is continuous with the pia mater. Astrocytes in the portion of the UON adjacent to the choroid are heavily interdigitated as they link to the PPS (Fig 3).

**Fig 4:**
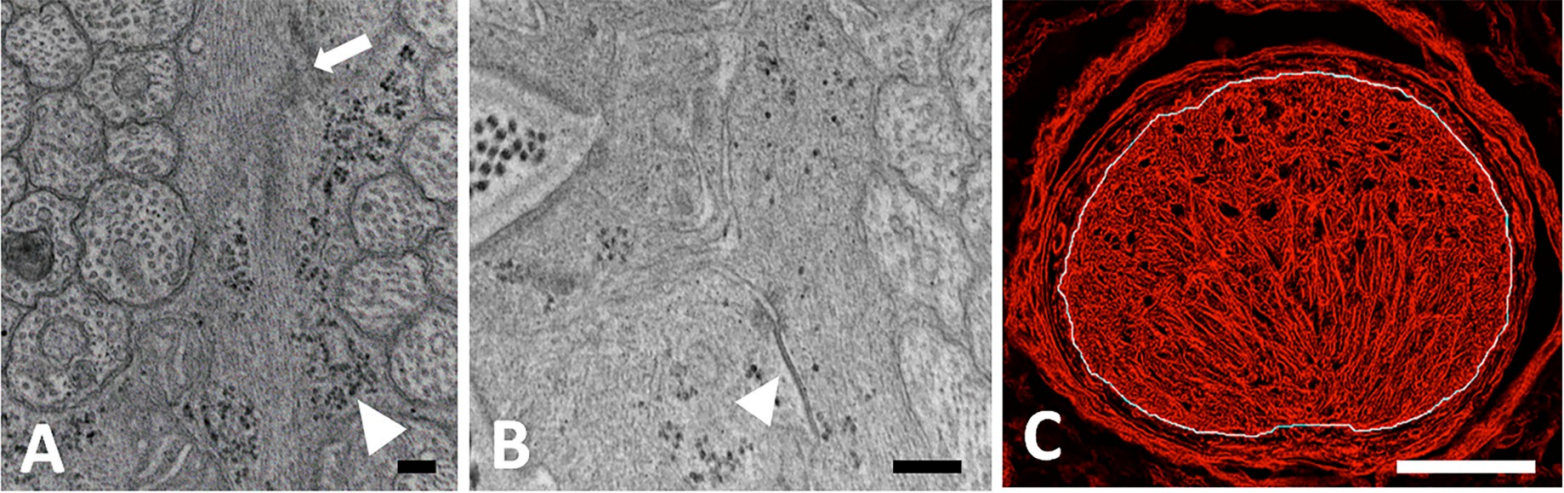
Intermediate filaments of astrocytes. A, B: TEM images of longitudinally sectioned, epoxy-embedded tissue from normal CD1 mouse unmyelinated optic nerve (UON). A: Normal astrocytes with intermediate filaments (arrow) and glycogen (arrowhead); B: Gap junction between astrocyte processes (arrowhead). C: Laser scanning micrograph of GLT1-GFP control mouse astrocyte processes labeled for actin (red) within the UON, its limit indicated in the image by white line. Scale bars A,B = 400 nm; Scale bar C = 100 μm.

The UON astrocyte cytoplasm is densely filled with a cytoskeleton containing both actin (Fig 4) and intermediate filament (GFAP) networks (Fig 3). There are rough endoplasmic reticulum, free ribosomes, and frequent glycogen granules. Many gap junctions join the cell membranes of astrocytes and there are occasional punctate adherent junctions between them. The capillary endothelial cells of the entire optic nerve have tight junctions, their own BM and sparse collagenous connective tissue with occasional fibroblasts. This vascular unit is everywhere lined by astrocytes and their BM.

### Histological alterations with elevated IOP

#### Naïve and contralateral controls

**Fig 5:**
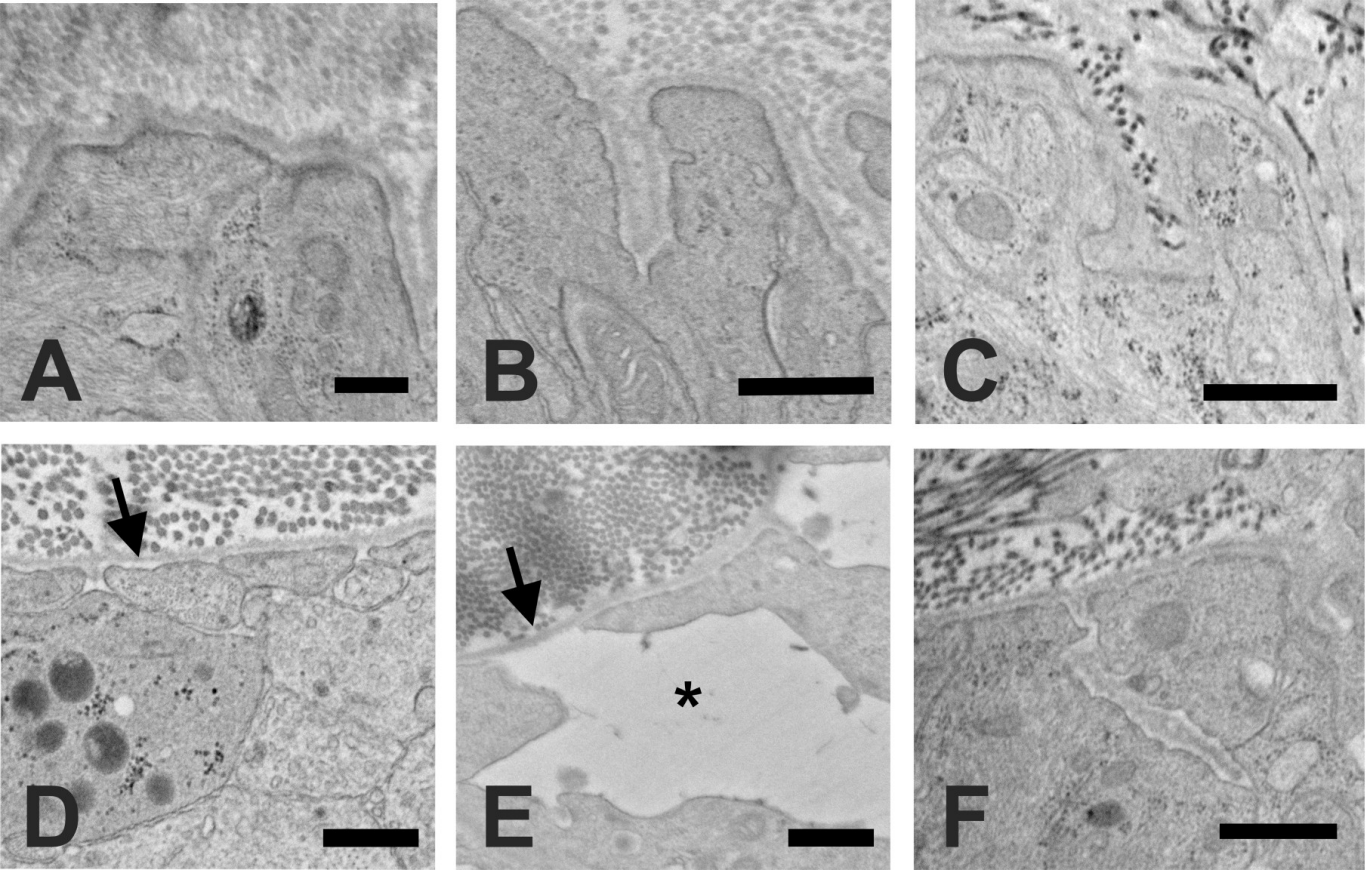
Astrocyte alterations after IOP elevation. Normal ONH astrocytes have electron dense junctions at their internal cell membrane facing the basement membrane (BM) in A: naïve (GLT1-GFP) and B: contralateral control (CD1) samples. C: After 1 day of elevated IOP, the junctional complexes were intact and unaffected (GLT1-GFP). D: After 3 days of elevated IOP, intracytoplasmic junctional complexes were absent from some areas of the portion of the plasma membrane facing the BM (arrow, GLT1-GFP). E: After 1 week of elevated IOP, junctional complexes continued to be only intermittently present and astrocytes were separated from each other, leaving an expanded extracellular space (asterisk) and areas of the BM that were bare and uncovered by astrocytes (arrow, CD1). F: After 6 weeks of elevated IOP, dense junctional complexes had reformed facing the BM and the extracellular spaces between astrocytes had returned to minimal spaces, as in controls (GFP-GFAP). Scale bars: A,B,D = 500nm; C,F = 800nm; E = 600nm.

After IOP elevation, we studied with TEM both bilaterally untreated (naïve) controls and contralateral control eyes of mice with unilateral bead injection in the fellow eye. Both naïve GLT1-GFP and GFP-GFAP mice (Fig 5A) and fellow control tissues (Fig 5B) had similar dense junctional complexes at the astrocyte/ peripapillary sclera border as well as similar astrocyte process orientation at this zone.

The histological findings after IOP elevation are presented in temporal sequence after 1 day, 3 day, 1 week and 6 weeks of glaucoma induction.

#### One day IOP elevation

After 1 day of elevated IOP, the ultrastructure of astrocytes was not noticeably changed, nor was there any change to the overall optic nerve appearance (Fig 5C). There was some early intra-axonal accumulation of vesicles and mitochondria, indicating axonal transport obstruction.

#### Three days IOP elevation

**Fig 6:**
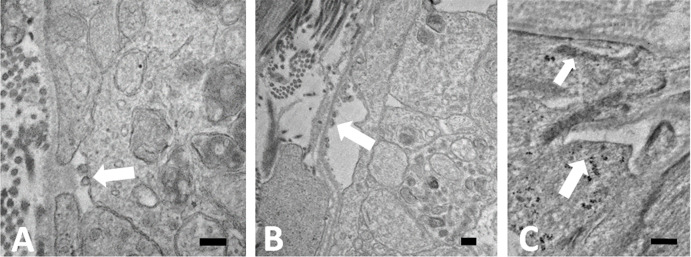
Loss of astrocyte adherence to BM. TEM images of longitudinal, epoxy sectioned tissue from 3 day IOP elevation GLT1-GFP mice in the unmyelinated optic nerve. A: After 3 days of elevated IOP, astrocytes separated from the basement membrane (BM) and small membrane blebs (arrow) appeared, either on the astrocyte external surface, or in B: membranous blebs were adherent to the BM. Vesicle and mitochondrial buildup due to transport block was seen in axons. C: Areas in which astrocytes normally have electron dense junctions at their internal cell membrane facing the BM either lacked this feature (larger arrow) or it was abnormally separated from the cell membrane (smaller arrow). Scale bars = 200 nm.

After 3 days of IOP increase, there were areas of abnormal extracellular space between astrocytes and surrounding axons, especially near the PPS in UON, that were not present normally (Fig 6A and 6B). These were large enough to be seen in light microscopy. Some segments of BM facing the PPS were abnormally devoid of astrocyte adherence. In such areas, some astrocyte cell membranes had small blebs of membranous material attached to them, or membrane blebs were left attached to the BM (Fig 6A and 6B). Where astrocytes had apparently withdrawn from their BM, the dense intracytoplasmic junctional complexes were separated from the plasma membrane or were altogether absent (Fig 6C). At 3 days after IOP elevation, axonal transport blockage was prevalent in many axons throughout the nerve, as evidenced by accumulation of vesicles and mitochondria with axon swelling (Fig 6B). After 3 days of IOP elevation, we quantified tissue rearrangements in UON, MT and MON, using longitudinal, light microscopic sections labeled for myelin basic protein to identify the MT position. In normotensive eyes, the mean MT position was 228.7 ± 32.7 μm (mean ± standard deviation) posterior to Bruch’s membrane opening, but was displaced to 288.8 ± 40.9 μm posteriorly after 3 day IOP elevation, indicating a substantial reorientation and reorganization of ONH tissue (t test, p = 0.019, n = 6 pairs of eyes).

#### One week IOP elevation

**Fig 7:**
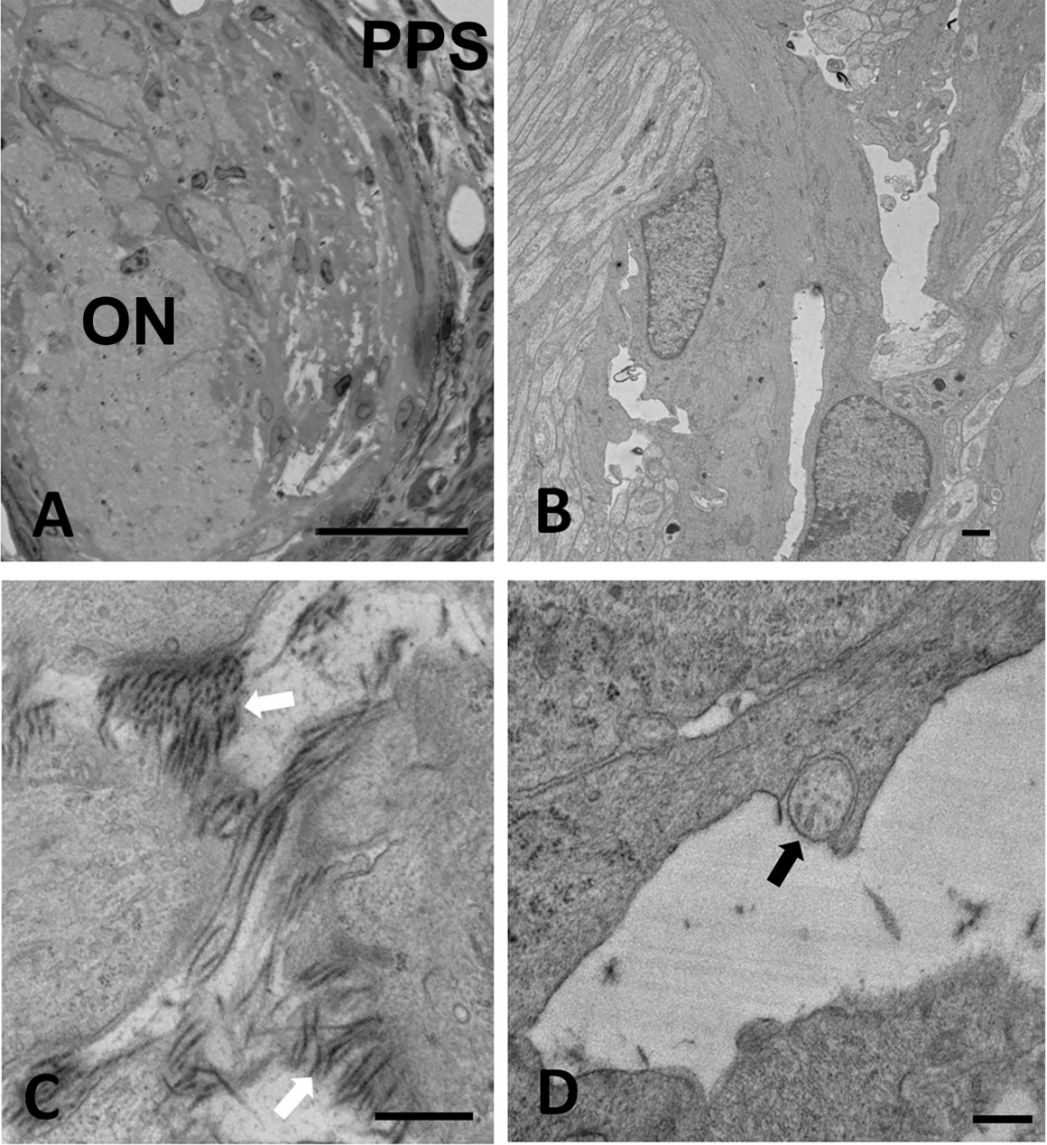
Astrocyte remodeling after 1 week IOP elevation. Images of longitudinal epoxy sectioned tissue from 1 week day glaucoma exposed CD1 mice. A: Epoxy stained cross-section of the optic nerve (ON) where abnormal spaces between astrocytes were found in more peripheral ON near the peripapillary sclera (PPS). B: Axons coursed longitudinally (vertical in this micrograph) out of the eye, to be oriented parallel to long axis of axons. Abnormal clear spaces were seen between astrocytes. C: In center of unmyelinated optic nerve there were newly formed collagen fibrils (arrows). D: Axons were often bare to the large extracellular spaces, compared to the normal situation in which they were covered by astrocytes (arrow). Scale bars: A = 25μm, B,C,D = 500nm.

**Fig 8:**
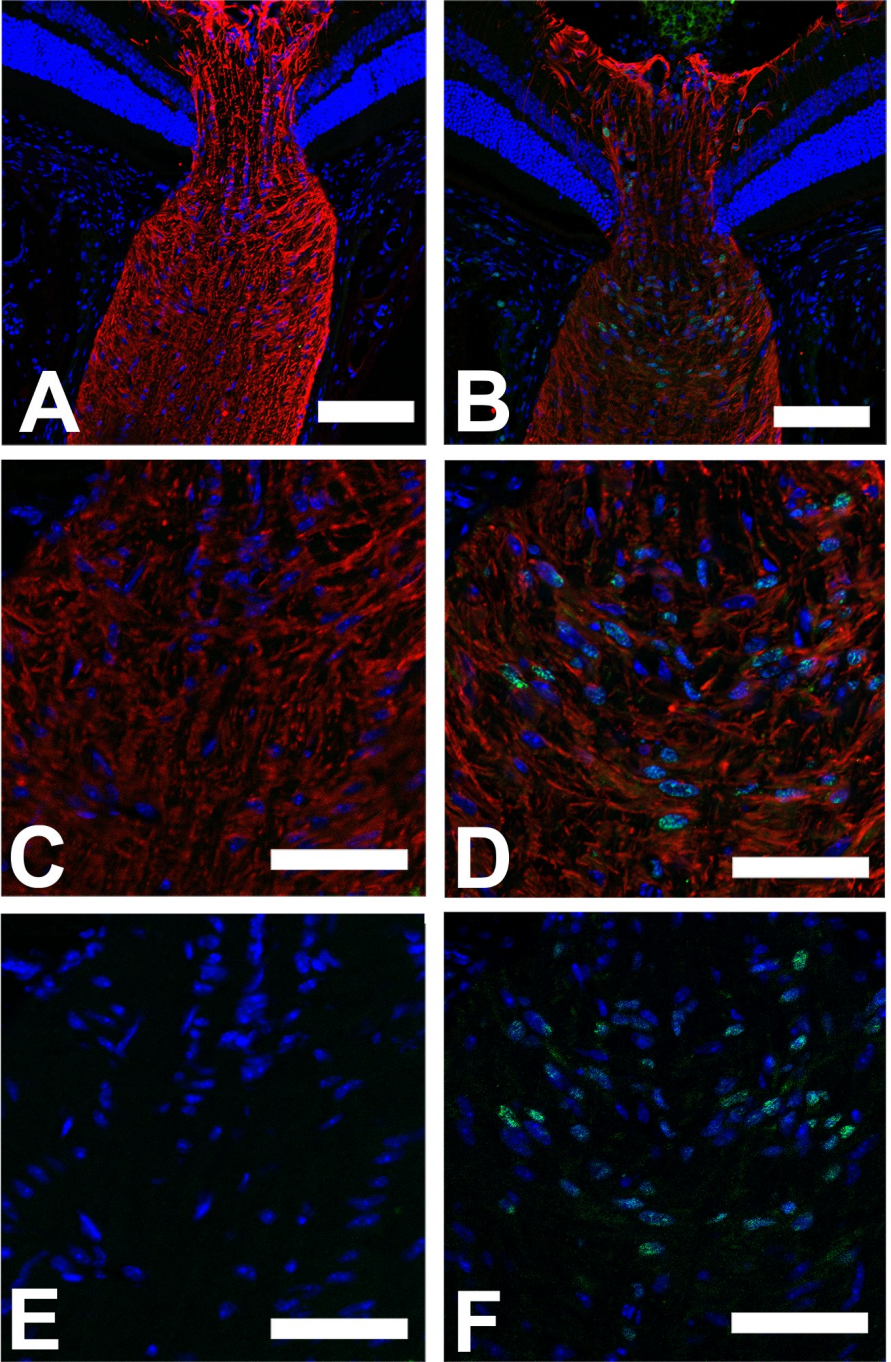
Ki67 label increase after IOP elevation. Longitudinal cryopreserved sections of the optic nerve in CD1 mice, labeled with DAPI (blue) and antibody to glial fibrillary acidic protein (GFAP, red) and to Ki67 (green), an indicator of cell proliferation. GFAP expresses the intermediate filament protein network of the astrocytes in all six images. Ki67 labeling was not visible in normal ONH (A,C,E), but after 3 day IOP elevation (B,D,F), substantial Ki67 labeling was visible in nuclei of astrocytes positive for GFAP in their cytoplasm, most abundantly in the unmyelinated optic nerve (see Table 4). E, F: DAPI and Ki67 label alone, showing greater positivity after IOP elevation. Scale bars: A, B = 100 μm; C, D,E,F = 50 μm.

After 1 week of IOP increase, there was a substantial reorientation of large and medium astrocyte processes in UON. Areas of abnormal open extracellular space were much increased over 3 day specimens, extending into the central portion of UON and visible by light microscopy (Fig 7A). While processes normally course across the ONH, the processes were shifted parallel to the axons (Fig 7B), consistent with the quantitative movement of the position of the MT measured above. In the expanded extracellular spaces (Fig 7B and 7D), there were newly formed collagen fibrils (Fig 7C). Axons in some areas had no astrocyte processes segregating them from the expanded extracellular space (Fig 7D). Some axons continued to have major vesicle and mitochondrial accumulation, while others were clearly degenerate. We also examined evidence for cell division and cell death among astrocytes by Ki67 labeling and TUNEL technique, respectively. There was substantial increase in nuclear Ki67 label at 3 days and one week after IOP elevation among astrocytes in the UON, the MT, and MON compared to contralateral controls (for all 3 mouse strains studied, Table 4 and Fig 8). By 6 weeks after IOP elevation, Ki67 labeling was the same as controls. Interestingly, Ki67 positivity was also evident in PPS fibroblasts. TUNEL labeling indicated only scattered, positive astrocytes, 1–2 per section, below the number for accurate quantification. Only occasional astrocytes undergoing apoptosis were seen by TEM (S1 Fig).

**Table 4:** Ki67 positive astrocytes after IOP elevation.

	Ki67 positive cells/mm^2^								
	CD1				C57BL/6			GLT1-GFP	
Region	CONTROL	3 DAY	1 WEEK	6 WEEKS	CONTROL	3 DAY	1 WEEK	CONTROL	3 DAY
Pre-Lamina	0.02±0.02	0.12±0.06***	0.04±0.03	0.04±0.01	0.003±0.01	0.44±0.31***	0.07±0.04*	0.00±0.00	0.08±0.10
Unmyelinated ON	0.00±0.00	0.52±0.37***	0.25±0.26	0.00±0.00	0.01±0.00	1.00±0.44***	0.32±0.22*	0.00±0.00	0.23±0.06*
Myelin Transition Zone	0.00±0.01	0.17±0.09**	0.18±0.08**	0.00±0.00	0.003±0.01	0.63±0.34***	0.32±0.09***	0.00±0.00	0.33±0.07**
Myelinated ON	0.00±0.01	0.07±0.05**	0.10±0.07*	0.00±0.00	0.01±0.02	0.34±0.20 ***	0.19±0.10**	0.00±0.00	0.18±0.16
Number of samples	5	5	4	4	17	9	5	3	3

n = 3–5 eyes per group,

* p<0.05,

** p<0.01,

***p<0.001, t tests.

#### Six week IOP elevation

**Fig 9:**
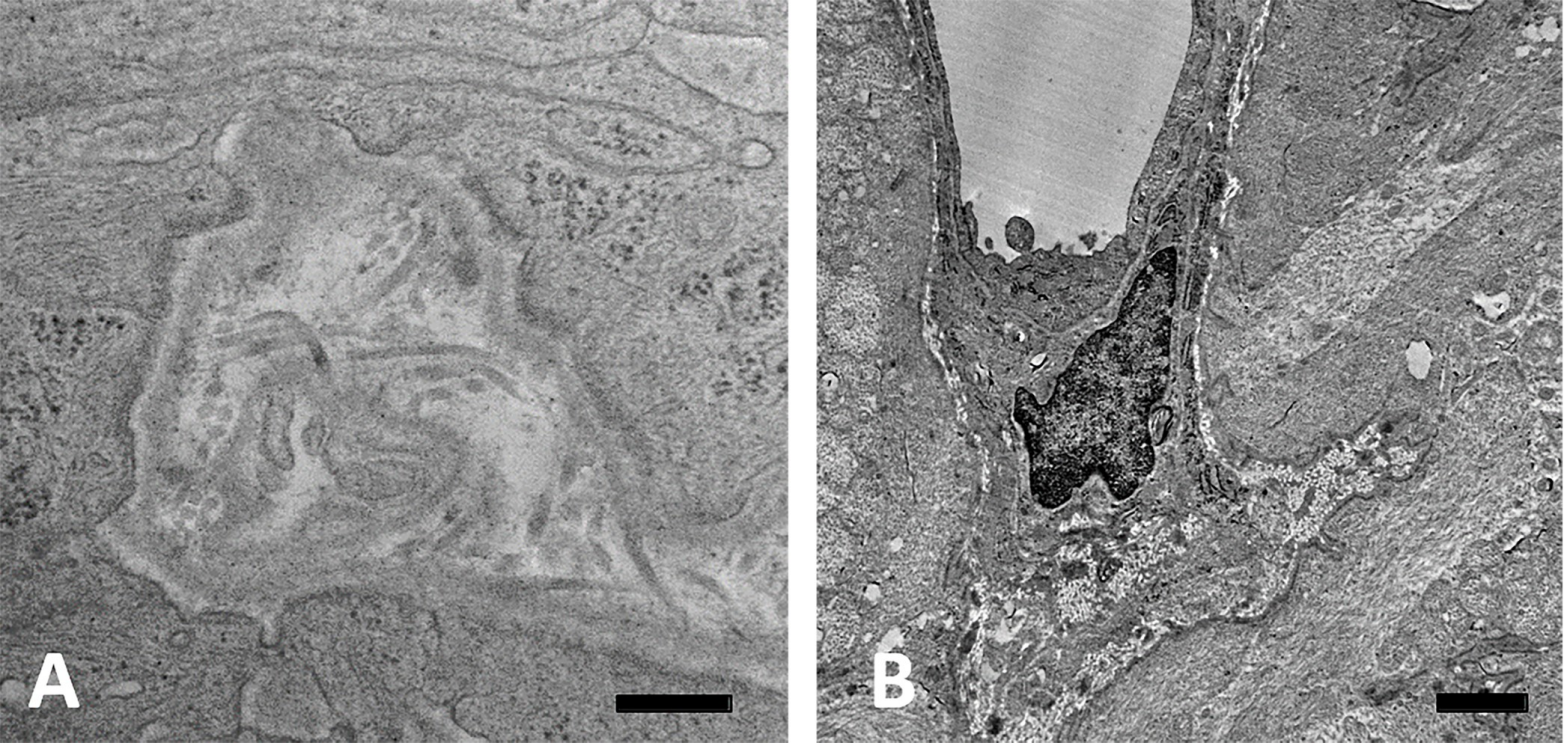
ONH structure at 6 weeks after IOP elevation. TEM images of longitudinal epoxy sectioned tissue from 6 week glaucoma exposed CD1 mice. A: Abnormal zone within the unmyelinated optic nerve (UON) shows new collagen formation and basement membrane (BM) formation. Astrocytes have reformed their dense junctional complexes facing the BM. B: Normal appearing capillary in UON with small amount of connective tissue, surrounded by BM of astrocytes, which line the vascular unit. Scale bars: A = 400 nm, B = 1 μm.

Six weeks after IOP elevation, there was only minimal remaining abnormal, open extracellular space in the UON (Figs 5F and 9). Some areas of abnormal, new collagenous connective tissue had formed (Fig 9A). At no time point did we detect a change in the appearance or number of ONH capillaries. Capillary endothelium and pericytes showed no abnormality throughout the entire period, including the retention of their tight junctions. The loss of RGC at 6 weeks was judged by axon counts and averaged from 15% loss in GLT1-GFP and CD1 mice to 66% in GFP-GFAP mice (n = 5–7 glaucoma eyes per group, p = 0.0001 for GFAP-GFP mice). The interstrain differences and absolute loss of axons are presented to indicate that some permanent damage occurred in each group by 6 weeks. We have previously reported much more extensive comparisons of axon loss by mouse strain in this model with much larger sample sizes [32].

### Junctional complex components

**Fig 10:**
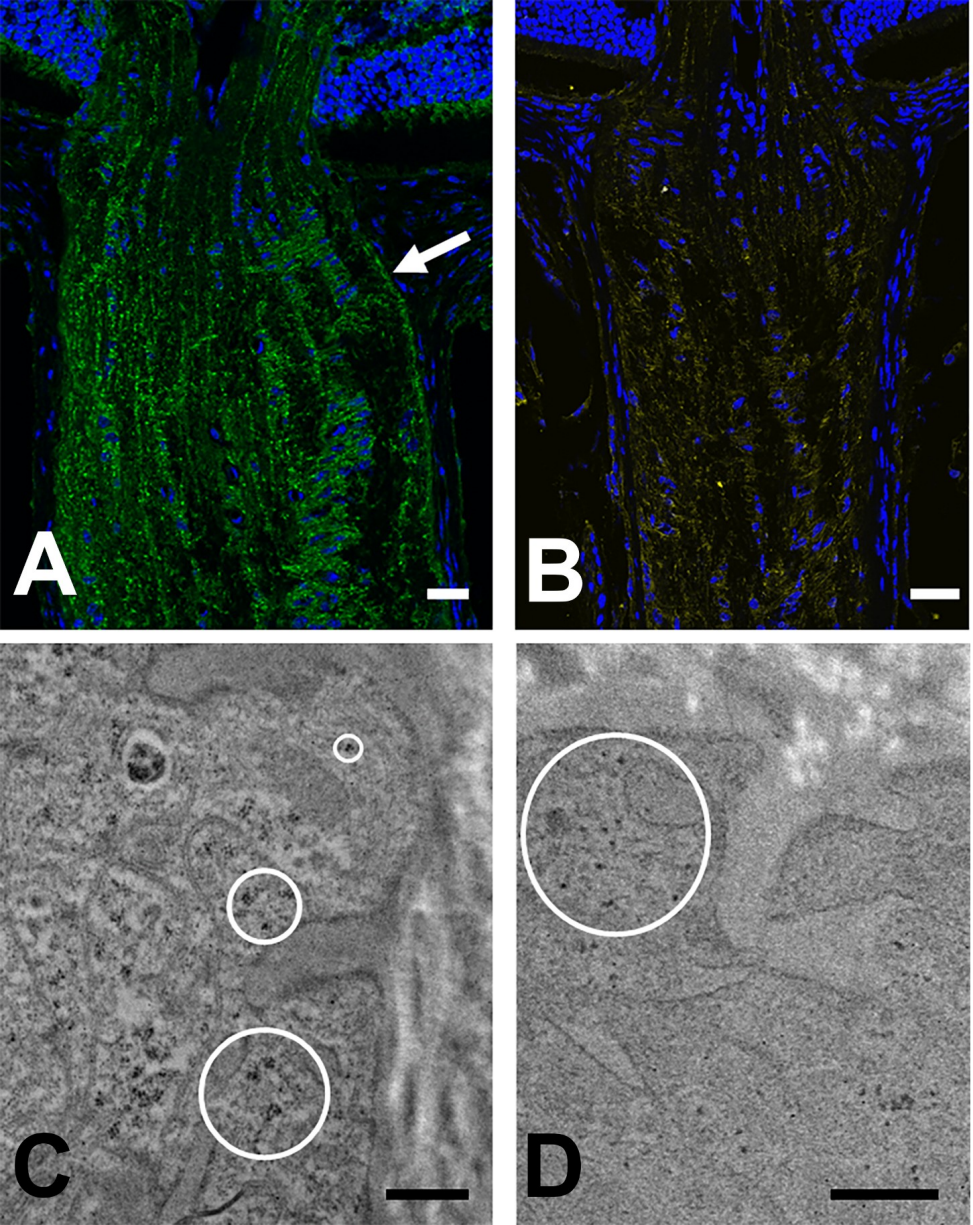
Immunolabeling of integrin β1 and α-dystroglycan. A & B: LSM images of longitudinal sections from CD1 mouse, immunolabeled for integrin β1 (A, green), α-dystroglycan (B, yellow) and DAPI (blue). Integrin β1 fluorescence in astrocytes was present throughout the optic nerve and was especially prominent at the peripapillary sclera (arrow). C & D: TEM images from 1 week IOP elevation GLT1-GFP mice in the unmyelinated optic nerve head. C: Gold particles (dark dots within circles) decorated antibodies to integrin β1 in astrocyte processes at junctions with PPS; D: Immunogold particles for α-dystroglycan were found in the most peripheral processes of astrocytes (circle). Scale bars: A, B = 50 μm; Scale bars C,D = 500 nm.

Integrin β1 prominently labeled astrocytes of the entire optic nerve, as well as the internal limiting membrane of the retina, areas surrounding blood vessels, and photoreceptor outer segments. While label was seen in astrocyte processes throughout, it was most prominent at the border zone with PPS (Fig 10). Immunolabeling of α-dystroglycan was similarly found in astrocytes throughout the PL, UON, MT and MON and in PPS fibroblasts (Fig 10). α-dystroglycan also was found surrounding the lumens of arteries and veins, while integrin β1 outlined the cellular component of vessel walls. α-dystroglycan was more intensely labeled toward the PPS border of astrocyte processes.

In the normal C57BL/6 and CD1 mouse ONH, astrocytes were labeled by immunogold-conjugated antibodies against integrin β1 and α-dystroglycan in both UON and MON and visualized by TEM (Fig 10C and 10D). Gold particles overlay the junctional complexes and were present in the terminal areas of astrocyte processes near to them. Labeling was minimal in portions of the astrocyte processes away from the terminal zone at the PPS.

## Discussion

### Unique phenotype of unmyelinated nerve astrocytes

UON astrocytes are subject to both hoop stress exerted by the PPS and translaminar pressure difference (IOP minus optic nerve tissue pressure). Two features that allow astrocytes in UON to support mechanical stress are the strength of their PPS attachment and their inherent resistance to stretching conferred by the cytoskeleton. These are shared by all mammalian ONH astrocytes, but, in larger animal ONH, LC collagenous beams reinforce the structure, as it would be impractical to have astrocytes bridging the entire nerve head, as they do in the smaller rodent ONH. The attachment of astrocytes to the PPS via their BM involves an intracellular junctional complex found by TEM in mouse, for the first time, which was previously observed in human nerve head astrocytes along their BM [33].

The PPS contains collagens type I and III and elastin [34], arranged circumferentially around the nerve head [35, 36]. The mouse PPS divides as it approaches the ONH into an inner portion that directly contacts astrocyte BM in the thinnest region of the UON and an outer portion that joins the pia mater, which also is separated from axons by astrocytes and their BM. Thus, IOP-generated hoop stress from PPS is transmitted throughout the UON, explaining why consequent axonal transport obstruction extends further posteriorly in mice and rats [37] than in humans. This unmyelinated segment is not present in pig, monkey or human ONH, in which myelination begins at the posterior lamina cribrosa border. Indeed, the astrocytic changes in this report were seen throughout the mouse UON, up to 250 μm posterior to the posterior scleral surface. These were not substantially different across the 4 strains of mice tested, including albino, agouti, and pigmented mice. The MON in mouse, as in other mammals, is not affected by acute transport obstruction and remains normal in appearance until actual Wallerian degeneration begins. At the region where UON contacts inner sclera and choroid, normal astrocyte processes and their BM are heavily interdigitated, increasing surface area contact with the PPS. This unique phenotype of UON astrocytes may be an adaptation to the force applied to them by IOP via the sclera and the translaminar pressure gradient.

### Astrocyte junctional complexes are altered in glaucoma mice

The astrocyte attaches to its BM by cell membrane-bound linkage molecules, two of which, integrin β1 and α-dystroglycan, were identified here by LSM and immunogold TEM at the PPS zone. To our knowledge, this is the first recognition of the dystroglycan complex as a transmembrane component in ONH astrocytes. The normal astrocyte BM contains collagens type IV and V, laminin, and heparin sulfate proteoglycans [38, 39]. Laminins are α,β,γ heterotrimers, with laminin-111 (α1β1γ1) and laminin-211 known to be expressed in astrocyte BM [40, 41]. Extracellular, glycosylated α-dystroglycan and its membrane-spanning β subunit arise from a single gene product. Syntrophins are membrane-associated adaptor proteins that serve as a platform that recruits signaling complexes and structural proteins to the dystroglycan complex [42]. Surrounding rodent optic nerve capillaries is both BM and a modest collagenous connective tissue, segregated from axons by the astrocyte BM and cytoplasm [43]. The electron-dense, junctional zone in astrocyte cytoplasm was first identified by Anderson in human ONH [33] and was reported in monkey [44], rat [45], and in this report in mouse UON in all 4 mouse strains examined. Yet, its composition, complex structure and function, and its responses to IOP elevation in the ONH had not been described. Astrocytes [46] and many other cell types were known to form such junctions under some conditions [47]. These junctional complexes are not present in retinal astrocytes, which are not subjected to similar mechanical stress, and they are uniquely present in UON astrocytes, but not in astrocytes of the MON. Membrane-bound integrin β1 and α-dystroglycan in these complexes bind cells to extracellular proteins by activating a conformational change in their ectodomain [48]. Both molecules are configured as heterodimers, whose interaction with extracellular laminin and agrin causes mechanotranslational changes, such as process reorientation and actin cytoskeletal changes [49].

The actions initiated by transmembrane integrin and dystroglycan translate external stress into alterations in actin and IF cytoskeletons [50] by intracellular binding of talin to promote actin filament bundling [51] and activity of focal adhesion kinase (FAK), Rho-family G-proteins, and scaffolding components: vinculin, paxillin, and zyxin [52]. These components and their translational pathways vary among cell types and conditions. Change in junctional complex components of optic nerve astrocytes in glaucoma models has only been recently studied. After recovery from acute IOP elevation, rat ONH tissues showed a decrease in phosphorylated FAK levels and increases in phosphorylated paxillin and cortactin, a downstream target of Src kinase [53]. Actin stress fibers consist of filaments cross-linked at one end by α-actinin and connected by myosin motors that generate isometric force by phosphorylation of myosin light chain. Inhibition of myosin or Rho kinase can lead to the cessation of actin fiber extension [54]. Mechanical strain enhances recruitment and activation of FAK by autophosphorylation, which stimulates RhoA and ROCK. In fibroblasts, this is known to promote the myofibroblast transition [55] with increase in αSMA.

We also observed that the astrocyte junctional complex detached from the cell membrane and was widely absent 1 week after IOP elevation, coincident with astrocytic separation from their BM. Bleb-like structures on the exterior surface of astrocyte surface may represent membrane fragments that were torn from cell attachment zones. Both findings indicate a loss of normal astrocyte linkage to PPS. In experimental rat glaucoma, extracellular matrix components increase in optic nerve head [56], and our observations are compatible with exposure of adjacent choroid and sclera to axons due to loss of the astrocytic barrier function. The lack of astrocyte coverage of the PPS zone shown here may explain the expansion of the extracellular space, since astrocytes normally prevent direct contact between RGC axons and the connective tissues and capillaries. While there are no intercellular tight junctions blocking fluid and solute movement, the position of astrocytes and their linkage to each other by gap junctions is ideal for management of the extracellular space. Many gap junctions were evident in mouse astrocytes, as in primates, serving as intercellular electrical and chemical coupling structures [57]. Interestingly, cell—cell focal adherens junctions were relatively sparse in normal mouse ONH astrocytes. By 6 weeks after IOP increase, the junctional complexes were present normally along cell membranes facing PPS throughout the unmyelinated zone and the zones of open extracellular space were gone.

The changes in astrocytic attachment to the PPS would likely alter ONH mechanical support, implying an early change in strain, prior to any matrix and astrocyte remodeling. An early change in ONH compliance was observed in monkeys 4 weeks after IOP elevation [58], and histology of monkey eyes in this phase showed lamina cribrosa displacement and scleral canal widening [59–64]. We have recently studied the biomechanical response of the mouse UON with short term IOP elevation. Differential strain in the peripheral compared to the central UON was seen 3 days after IOP increase, consistent with the present disconnection of ONH astrocytes from PPS (unpublished observations).

### Mechanotranslational behavior of astrocytes in glaucoma

We found rapid astrocyte cell division 1 week after IOP elevation, as also reported in rat glaucoma [65]. A general retraction of astrocytes from PPS followed by complete astrocyte loss was reported in one rat glaucoma model [66]. This finding was contradicted by abundant astrocyte division found in another rat glaucoma model [67]. In mouse glaucoma and in a monkey glaucoma model [68], there was minimal astrocyte death, as indicated by only occasional apoptotic ultrastructure and TUNEL labeling. Coincident with cell division, the remodeling translational responses of astrocytes included new collagen synthesis and active phagocytosis of axonal debris. In experimental monkey glaucoma, ONH connective tissue volume initially increased [69]. In human glaucoma, astrocytes migrate into LC pores, form layers on the LC surface and fill spaces abandoned by dying RGC axons [70]. Active phagocytosis [71], as well as production of new collagens types I, III, IV, and VI was seen after experimental mouse [72, 73], monkey, and human glaucoma [74]. This remodeling was seen in experimental monkey glaucoma, but not after optic nerve transection in the monkey, and thus is a specific astrocytic reaction to IOP-induced changes. Jakobs et al [69] noted that open spaces present soon after IOP elevation had disappeared 4 weeks later. Likewise, our 6 week specimens had no remaining abnormal extracellular space, despite significant loss of axons.

A second feature of astrocyte remodeling was the reorientation of processes from across the UON to parallel to axons, previously documented in mouse and rat [75] glaucoma. This rearrangement in our observations was a displacement of existing processes, while Jakobs et al. have additionally found longitudinally oriented new processes after prolonged IOP elevation [76]. In parallel research, we are quantifying the number and orientation of astrocyte processes identified by actin and GFAP labeling in mouse glaucoma. These data confirm a shift in process orientation toward the long axis of the nerve. This outward redirection of existing astrocyte cell bodies and processes is compatible with the displacement away from the eye of the MT as quantified here, in apparent response to the increased translaminar pressure difference. We have shown [77] that the scleral canal widens naso-temporally in mouse experimental glaucoma [78], but its expansion is limited by PPS stiffness, subjecting the cellular mouse LC to stress longitudinally. Changes in overall glial coverage have been suggested in the MON of mice with glaucoma [79], though as described above, the phenotype of astrocytes in the UON is distinct from those in MON.

For cell processes to reorient, the internal cytoskeletal machinery must translate mechanosensitive signaling from the integrin and dystroglycan complexes into physical movements that are either beneficial, detrimental or both to overall axonal health. Gene array and immunofluorescence study of glaucoma monkey optic nerve found increased expression of transforming growth factor β (TGFβ) [80, 81]. Cultured human ONH fibroblasts (“lamina cribrosacytes”) respond to stretch by increased expression of thrombospondin 1 and TGFβ2 [82]. Our proteomic analysis of mouse glaucoma sclera and ONH showed increases in integrin-linked signaling, actin cytoskeletal signaling, and Rho-kinase pathway molecules, with specific increase in actinin, a major component of the junctional complex [29]. The increase in TGFβ signaling in these investigations in mouse glaucoma was accompanied by increases in the downstream factors, pERK and pSMAD [83]. The integrin mechanosensation pathway may liberate TGFβ from its latent, extracellular form, impacting RhoA and ROCK signaling and directly influencing changes in the actin cytoskeleton. Known effects of TGFβ signaling include features observed here, including cell division, myofibroblast-like transition, and collagen fibrotic response.

It has been typical to ascribe all astrocyte changes as “reactive” with the implication that they are all detrimental to neuronal survival and reestablishment of baseline functions. This has recently been shown to be too simple an assumption, with recognition of both protective and pathological astrocyte phenotypes [84]. Furthermore, experimental IOP elevation in mouse induces up-regulation and phosphorylation of transcription factor STAT3 in ONH astrocytes. Deletion of the STAT3 gene leads to increased RGC loss from experimental glaucoma, as well as reduced astrocyte process expansion [85]. In a rat glaucoma model, but not after optic nerve transection, we found gene expression increases in STAT1 and STAT3 [86]. In CNS astrocytes, the response to stretch is mitigated by STAT3 [87, 88] and its activation is dependent on ROCK stimulation in fibroblasts [89]. Investigations are needed to determine which astrocyte mechanotranslational pathways are beneficial and therefore represent potential therapeutic targets.

Some observations in this report, while made in many eyes and several types of mice, were qualitative, though they are supported by quantitative methods shown here and in upcoming publications. Due to the nature of TEM tissue preparation, dehydration could have altered the degree of open space; however, preparation conditions were identical for glaucoma specimens and for controls that lacked the abnormal extracellular space. The location of molecules labeled by gold particle—antibodies is not exact, as the TEM methods involve the position of primary antibody, secondary antibody and the attached gold particle. The degree of axon loss at 6 weeks included insufficient numbers of eyes to produce robust estimates of interstrain differences in loss of axons. We have reported more extensive comparisons of axon loss by strain with this model in much larger sample sizes [32].

### Summary

Chronic IOP elevation leads to significant changes in UON astrocytes relevant to their mechanosensation mechanisms. Junctional complex densities adjoining their BM are altered after 1 week of IOP increase, associated with astrocyte separation from their attachment to the PPS. Astrocyte processes reorient along the long axis of the optic nerve and increased extracellular space develops at 1 week after IOP increase. Resolution of open spaces is coincident with abnormal, new collagen formation as part of tissue remodeling. Further study of the mechanotransduction of external stress into astrocyte responses, including αSMA expression in glaucoma is merited.

## Supporting information

S1 Fig Apoptotic cell nucleus. TEM images from 1 week IOP elevation GFP-GFAP mice. A: Apoptotic cell nucleus of an astrocyte with loss of nuclear membrane. B. Astrocyte in process of apoptosis with clumping of chromatin. Scale bar = 800 nm.
(TIF)

S1 Table Overview of experiments. (DOCX)
