# Supplementary figures and images for: Astrocyte responses to experimental glaucoma in mouse optic nerve head
Source: PLoS One. 2020 Aug 21;15(8):e0238104. doi: 10.1371/journal.pone.0238104 (PMC7442264; doi:10.1371/journal.pone.0238104)

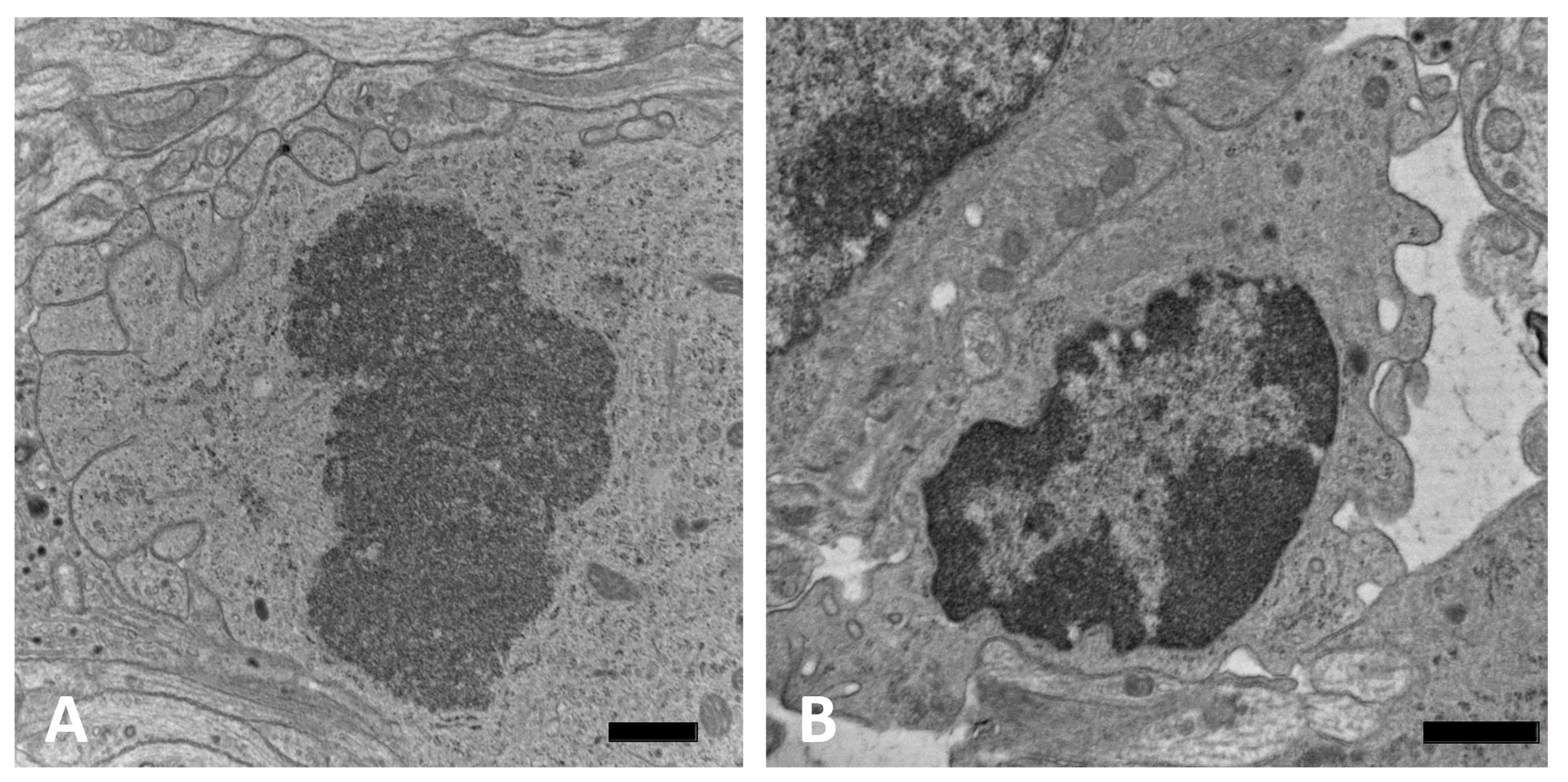

Supplement: S1 Fig — TEM images from 1 week IOP elevation GFP-GFAP mice. A: Apoptotic cell nucleus of an astrocyte with loss of nuclear membrane. B. Astrocyte in process of apoptosis with clumping of chromatin. Scale bar = 800 nm. (TIF) [file pone.0238104.s001.tif]
