# Supplementary material for: Astrocyte responses to experimental glaucoma in mouse optic nerve head
Source: PLoS One. 2020 Aug 21;15(8):e0238104. doi: 10.1371/journal.pone.0238104 (PMC7442264; doi:10.1371/journal.pone.0238104)
Supplement: S1 Table — (DOCX) [file pone.0238104.s002.docx]

**S1 Table: Overview of Experiments**

| **Experiment** | **Primary used** | **Secondary used** | **Diluents** | **Primary incubation time** | **Secondary incubation time** | **DAPI incubation time** | **Controls used** |
| --- | --- | --- | --- | --- | --- | --- | --- |
| Actin | Anti-phalloidin | None | PBS* | 1 hour | None | 10 Minutes | Cell cytoplasm |
| Glial fibrillary acidic protein (GFAP) | Anti-glial fibrillary acidic protein (GFAP) | Laser 647 | PBT**+ 0.1%BSA^ | Overnight | 1 hour | 10 Minutes | Astrocytes from unmyelinated optic nerve |
| Ki67 | Anti-Ki67 | Laser 488 | PBT**+ 0.1%BSA^ | Overnight | 1 hour | 10 Minutes | Stressed tissue- glaucoma samples |
| Myelin basic protein (MBP) | Anti-myelin basic protein (MBP) | Laser 488 | PBT**+ 0.1%BSA^ | Overnight | 1 hour | 10 Minutes | Myelinated portion of optic nerve |
| α-dystroglycan | Anti-α-dystroglycan | Laser 488 | PBT**+ 0.1%BSA^ | Overnight | 1 hour | 10 Minutes | Extracellular component of muscle and nerve |
| Integrin β1 | Anti-Integrin β1 | Laser 488 | PBT**+ 0.1%BSA^ | Overnight | 1 hour | 10 Minutes | Blood vessels |
| Immunogold- α-dystroglycan | Anti-α-dystroglycan | Goat-anti-rabbit IgG (H&L) ultra gold | PBT**+ 0.1%BSA^ | Overnight | Overnight | N/A | External eye muscles |
| Immunogold-Integrin β1 | Anti-Integrin β1 | Goat-anti-rabbit IgG (H&L) ultra gold | PBT**+ 0.1%BSA^ | Overnight | Overnight | N/A | Blood vessels |

*PBS=Phosphate Buffer Saline, **PBT= PBS+0.25% Triton-X100, ^BSA=Bovine Serum Alubumin
